# Policies to increase the social value of science and the scientist satisfaction. An exploratory survey among Harvard bioscientists.

**DOI:** 10.12688/f1000research.3-20.v2

**Published:** 2014-06-06

**Authors:** Andrea Ballabeni, Andrea Boggio, David Hemenway

**Affiliations:** 1 Department of Health Policy and Management, Harvard School of Public Health, Boston, MA, 02115, USA; 2 Department of History and Social Science, Bryant University, Smithfield, RI, 02917, USA

## Abstract

Basic research in the biomedical field generates both knowledge that has a value *per se* regardless of its possible practical outcome and knowledge that has the potential to produce more practical benefits. Policies can increase the benefit potential to society of basic biomedical research by offering various kinds of incentives to basic researchers. In this paper we argue that soft incentives or “nudges” are particularly promising. However, to be well designed, these incentives must take into account the motivations, goals and views of the basic scientists. In the paper we present the results of an investigation that involved more than 300 scientists at Harvard Medical School and affiliated institutes. The results of this study suggest that some soft incentives could be valuable tools to increase the transformative value of fundamental investigations without affecting the spirit of the basic research and scientists’ work satisfaction. After discussing the findings, we discuss a few examples of nudges for basic researchers in the biomedical fields.

## Introduction

Basic or fundamental research—generally defined as untargeted research seeking to expand knowledge—is a key component of innovation. While it generates knowledge that has a value *per se* regardless of its possible practical outcome, it also delivers knowledge that has the potential to produce more practical benefits ^1, 2^. Basic biomedical research in particular is crucial in addressing the challenges we face in our highly interconnected planet in which communicable diseases spread quickly and in which non-communicable diseases cause the premature death of many individuals ^3^.

Historically, a wide range of basic biomedical research projects have contributed to the advancement of knowledge, from research solely inspired by the researcher’s curiosity to projects driven by a vision of how knowledge generated by research could be used as the basis for applied research. All research along this continuum is considered “basic” because it serves as the foundation for further research that may lead to applications. Scientific knowledge is produced by the coming together of all kinds of research streams and ideas. Abraham Flexner captured this aspect of science in the image of the Mississippi river, which *“begins in a tiny rivulet in the distant forest. Gradually other streams swell its volume. And the roaring river that bursts the dikes is formed from countless sources* ^4^ *”*. Although it often takes decades to develop, the applied outputs of knowledge advancement (e.g. drugs) have at their roots countless basic investigations ^2^.

Given its importance, complexity and breadth, basic research has been primarily funded by public money. This was particularly true in the decades that followed World War II during which basic research went through a “golden age,” being conducted primarily in research universities and paid for with public money ^5^. Sadly, public expenditure for research has decreased since then and nowadays fundamental sciences are for the most part underfunded. Basic biomedical research currently receives less support than it received only a few years ago. For instance, in the United States, National Institutes of Health (NIH) funding has been nearly stationary since 2003 in the face of rapid expansion (as increased amount of research activity) of existing biomedical fields and the emergence of new ones ^1, 6^. One explanation of the low support for fundamental sciences is our cognitive bias in favor of immediate rewards. As our brains are structured in a way that leads us to unduly favor immediate rewards over future benefits ^7^, we tend to underestimate the importance of human activities and initiatives with benefits that lie in the future ^8^.

Nowadays basic researchers are said to owe a moral duty to extract maximum transformative value (the potential to translate in novel and fruitful applied research) whenever their research is publicly funded ^9– 11^. The mindset has changed since the time when the isolation and self-referring of the scientific community was perceived in a positive way and when the concept of “scientific integrity” was equated with the concept of “social responsibility” ^12– 14^. Public support is no longer (or is much less) based on the myth of the “free play of free intellects” ^2, 12, 15^ and the notion of “socially robust” knowledge has often replaced the notion of “reliable knowledge” ^16, 17^. Attention is now more often focused on whether public funding is used for socially beneficial activities ^11, 18^. An increasing number of scholars and opinion leaders are proposing the training of “civic scientists” and the engagement of scientists in the public discourse ^19– 24^. Specific policies have been designed with the purpose of increasing the practical output of basic academic research. For example, the Bayh-Dole Act gives US universities the possibility to own their own inventions but it also raises concerns as to whether the monetary incentives are too strong and therefore distract basic scientists from focusing on fundamental questions ^25, 26^. Funding agencies such as the NIH, in order to assess and increase the transformative value of research, require the discussion of the health benefit potential or (social) “significance” of proposed research when assigning resources. However, some scholars think that the current system of peer reviewing and grant assignment stifles creativity and innovation ^6, 27^.

New strategies to successfully maximize the transformative value of basic research without compromising the nature of fundamental inquiries and the scientist’s creativity (and satisfaction) are needed. Softer incentives, not based on restrictive policies, which are often called by behavioral economists “nudges” ^28^, seem particularly promising. If properly designed for basic research, nudges would slightly (and sometimes unperceivably) reorient some scientists in a certain direction without imposing rules or decreasing work satisfaction. However, these “nudges” can be designed well for basic research only if we have a good grasp of what motivates basic scientists, what their values are and the intellectual frameworks in which they operate so that soft incentives can be properly tailored. Studies on scientist’s views and values often focus on research misconduct ^29– 31^ or on particular issues of specific biomedical fields ^32– 36^. A few studies have collected feedback from scientists about social responsibility. Most of these studies were based on interviews and focus groups; in a few cases surveys were used to collect feedback about specific issues ^12, 37– 39^. In this paper we expand upon the existing body of knowledge. We believe our study is the first based on a survey containing an extended set of multiple-choice and numerical questions aimed at quantitatively elucidating the motivations, values and opinions of a large group of basic researchers working in different fields of biomedicine. Data come from the responses of more than 300 basic scientists at Harvard Medical School and affiliated institutions in Boston and Cambridge, Massachusetts (USA).

 In this paper, we present the results of the survey and discuss how our findings can be used to increase the transformative value of basic biomedical research without decreasing the motivations and freedom of the scientists. We provide examples of specific nudges that might increase the social benefit without decreasing the “basic” nature of the scientific investigations.

## Results

### Description of the survey and the sample

The survey was an online questionnaire comprised of 17 questions (Q1–Q17). On average, fewer than 2% of the respondents, with a range from 0% (Q2 and Q3) to 6.3% (Q15) skipped any of the 17 questions. Answers could be provided through multiple choices or, alternatively, textboxes for alphanumerical entries (see Methods section for additional details). 304 scientists took the survey. The first four questions of the questionnaire (Q1–Q4) gathered data on the sample characteristics.

**Figure S1.**
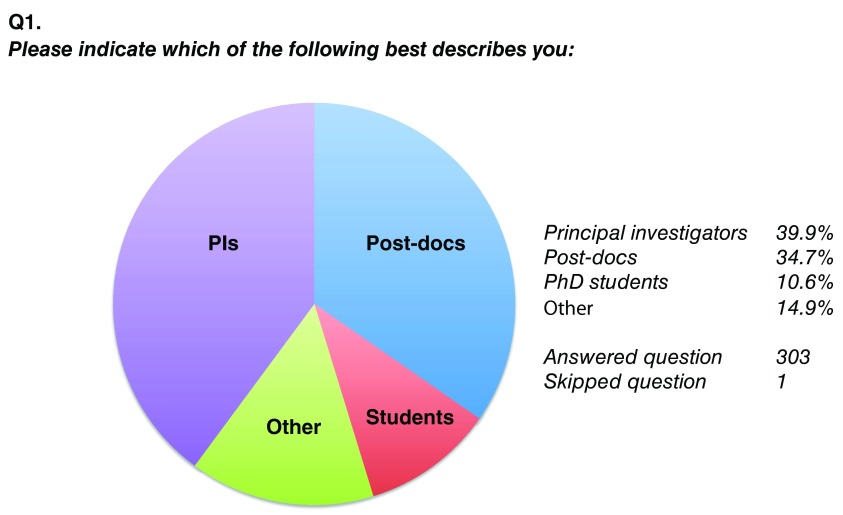
Academic positions of the surveyed scientists.

**Figure S2.**
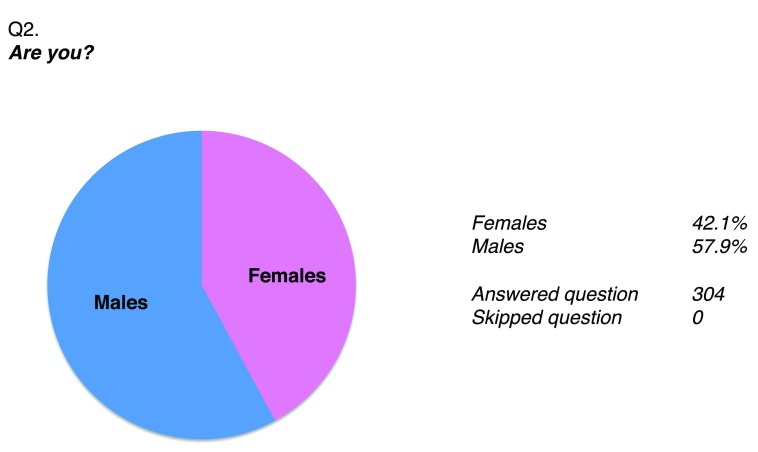
Gender distribution of the surveyed scientists.

**Figure S3.**
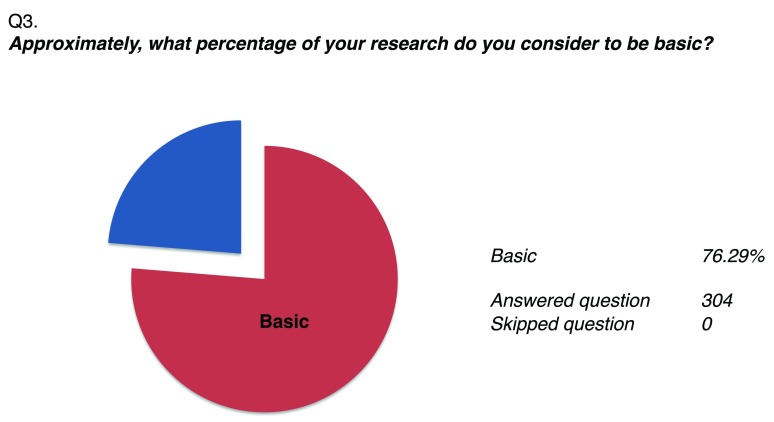
Level of involvement in basic research of the surveyed scientists.

**Figure S4.**
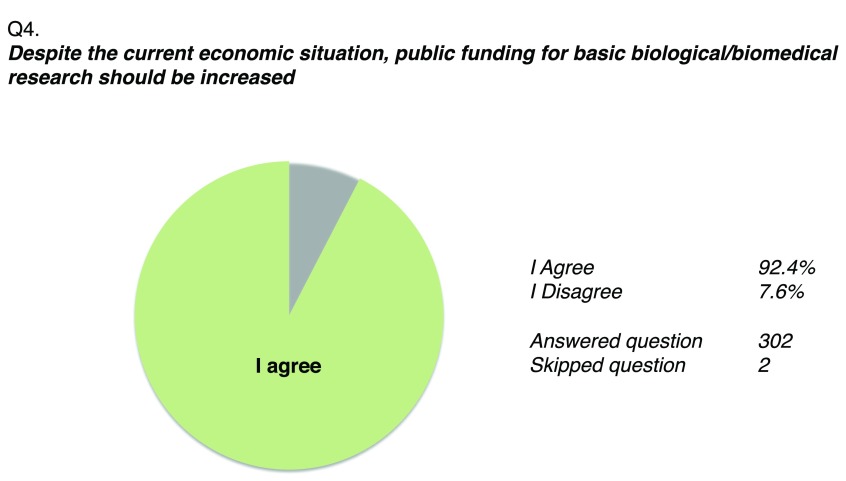
The vast majority of basic scientists think that public funding for basic biological/biomedical research should be increased, despite the current economic situation.

The first question (Q1) (all questions hereinafter will be referred to as Q#) aimed at identifying the respondent’s academic position: 39.9% were principal investigators, 34.7% post-docs, 10.6% PhD students and 14.9% belonged to other categories (including “research assistants” and “research technicians”) ( Figure S1). Among the respondents, 42.1% were females and 57.9% males (Q2) ( Figure S2). On average, respondents reported spending 76.3% of their research time on basic research (Q3) ( Figure S3), with only 3.6% of respondents stating that they were not involved (0%) in basic research. Q4 asked them if they agreed/disagreed with the following statement: *“Despite the current economic situation, public funding for basic biological/biomedical research should be increased”.* 92.4% of the respondents agreed while only 7.6% disagreed ( Figure S4). Overall, these results show that our purposive sample was well balanced with regard to academic position and gender, significantly involved in basic investigations and supportive of increased public funding for basic biological/biomedical research.

### Basic scientists think that considering the practical benefits of their research is compatible with the notion of basic science

**Figure 1.**
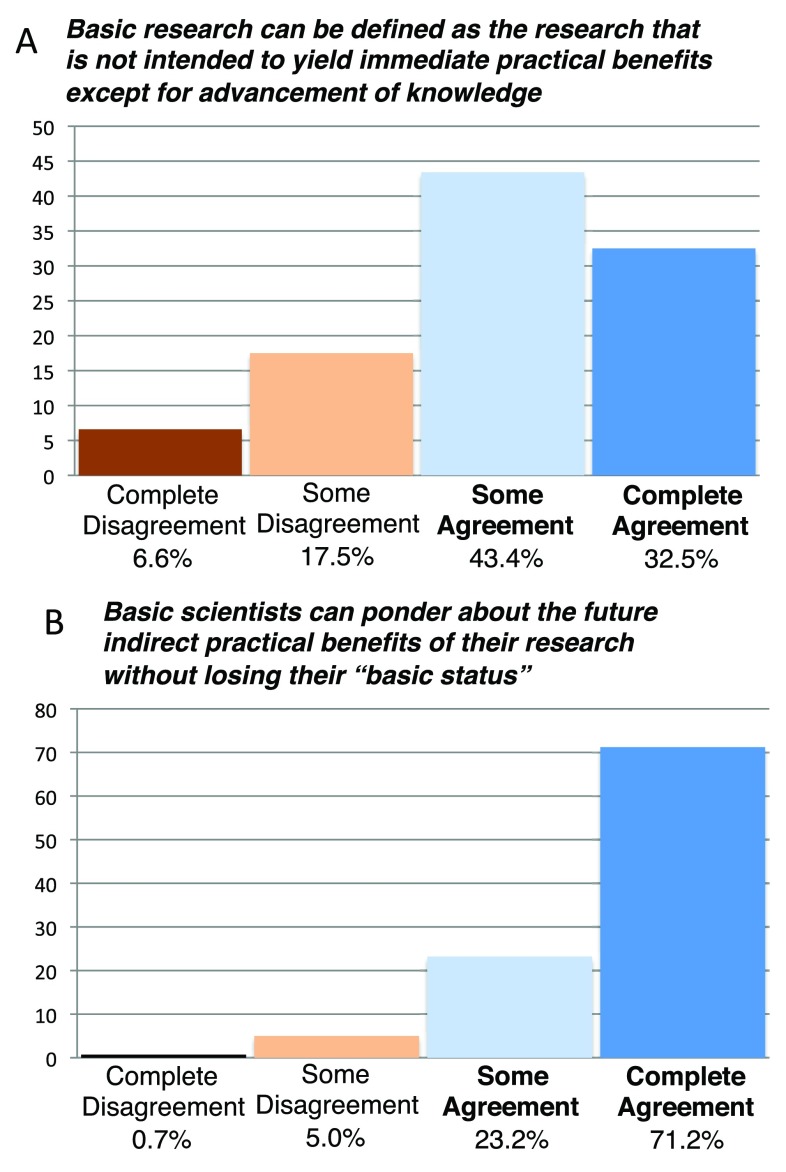
How do scientists conceptualize basic “bio” research? ( **A**) Graph shows the levels of agreement to the following statement: “Basic research can be defined as the research that is not intended to yield immediate practical benefits except for advancement of knowledge”. 302 scientists answered the question; 2 skipped. ( **B**) Graph shows the levels of agreement on the following statement: “Basic scientists can ponder about the future indirect practical benefits of their research without losing their “basic status””. 302 respondents answered the question; 2 skipped.

The way scientists conceptualize basic research is important not only to define the concept but also to design policies that can effectively promote it. Q5 asked respondents to express their level of agreement with the following: *“basic research can be defined as the research that is not intended to yield immediate practical benefits except for advancement of knowledge”*. Among respondents, 32.5% expressed complete agreement, 43.4% some agreement, 17.5% some disagreement and 6.6% complete disagreement ( Figure 1A). A complementary question (Q6) asked about the level of agreement with the following: *“basic scientists can ponder about the future indirect practical benefits of their research without losing their “basic status””*. 71.2% of the respondents expressed complete agreement, 23.2% some agreement, 5.0% some disagreement and 0.7% complete disagreement ( Figure 1B). These results indicate that most scientists surveyed think that considering the indirect practical outcome of basic scientific investigations is compatible with the notion of basic research. In other words, basic research should not be conceptualized as being necessarily (or solely) driven by curiosity.

### What is the goal of biological and biomedical research?

**Figure 2.**
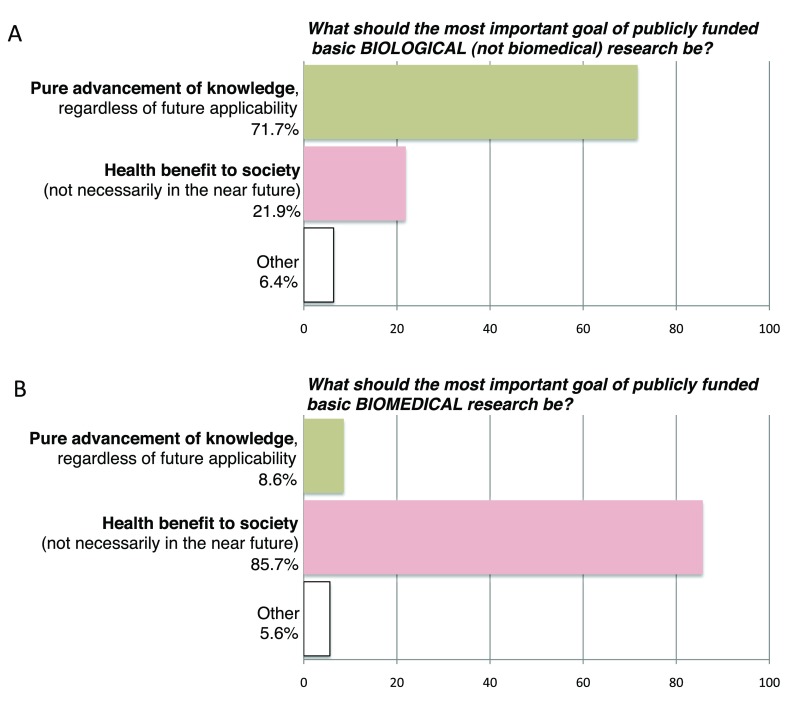
What is the main goal of biological and biomedical research? ( **A**) Graph shows how scientists answered the following question: “What should the most important goal of publicly funded basic BIOLOGICAL (not biomedical) research be?”. Surveyed scientists were given the indicated three choices. 297 answered the question; 7 skipped. ( **B**) Graph shows how scientists answered the following question: “What should the most important goal of publicly funded basic BIOMEDICAL research be?” Surveyed scientists were given the indicated three choices. 301 answered question; 3 skipped.

Q9 asked respondents to answer the following question: *“What should the most important goal of publicly funded basic BIOLOGICAL (not biomedical) research be?”* Of the three options, 71.7% responded “pure advancement of knowledge, regardless of future applicability”, 21.9% responded “health benefit to the society (not necessarily in the near future)” and 6.4% responded “other”, writing things such as the “environmental or economical benefit to society” or “sustainability of our species and of the biosphere” ( Figure 2A). Similarly, Q10 asked the following question: *“What should the most important goal of publicly funded basic BIOMEDICAL research be?”* Responses were different; only 8.6% of the respondents answered “pure advancement of knowledge regardless of future applicability”, while 85.7% answered “health benefit to society (not necessarily in the near future)” and 5.6% chose “other” ( Figure 2B). These results suggest that the scientists surveyed perceive the primary goals of “biological research” and “biomedical research” to be different, with a propensity to include “pure advancement of knowledge” as an important goal of “biological” research only.

### What motivates the basic scientists?

**Figure 3.**
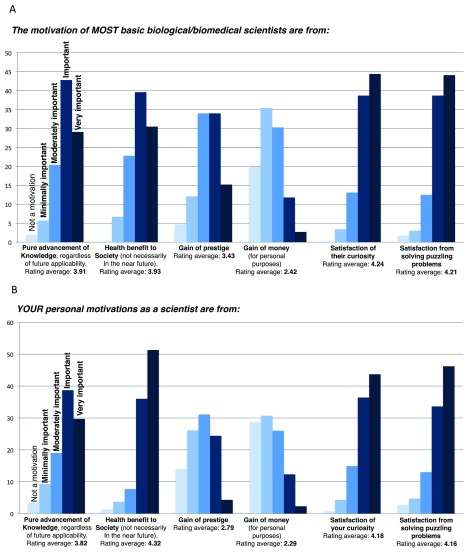
What does motivate the basic scientists? ( **A**) Graph shows the responses of the surveyed scientists to the following input: “the motivation of MOST basic biological/biomedical scientists are from:”. Six different types of motivations were proposed. Respondents could rate each type of motivation as “not a motivation, “minimally important”, “moderately important”, “important” or “very important”. Rating averages for each type of motivation are also indicated (the scores were 1 to 5, from “not a motivation” to “very important”). 299 respondents answered the question; 5 skipped. ( **B**) Graph shows the responses to the following input: “YOUR personal motivations as a scientist are from:”. Six different types of motivations were proposed. Respondents could rate each type of motivation as “not a motivation, “minimally important”, “moderately important”, “important” or “very important”. Rating averages for each type of motivation are also indicated (the scores were 1 to 5, from “not a motivation” to “very important”). 302 respondents answered the question; 2 skipped.

Understanding the motivations of people is important for designing policies that offer incentives to pursue certain goals. We therefore designed two questions to gather information on what motivates basic scientists. The respondents were asked to select their level of importance (“not a motivation”, “minimally important”, “moderately important”, “important” and “very important”) for six motivations. The rating average was then calculated by assigning a score from 1 to 5 to these options. Scientists were asked to provide feedback on (Q7) *“the motivations of most basic biological/biomedical scientists are from:”*. The rating average, for the motivation “pure advancement of knowledge, regardless of future applicability” was 3.91. The rating for “health benefit to society (not necessarily in the near future)” was 3.93. The rating for “gain of prestige” was 3.43. The rating for “gain of money” was 2.42. The rating for “satisfaction of their curiosity” was 4.24. The rating average for “satisfaction from solving puzzling problems” was 4.21 ( Figure 3A).

To see if scientists perceive themselves differently from other scientists, we also asked respondents to provide feedback on the following input (Q8): *“YOUR personal motivations as a scientist are from:”.* The rating for “pure advancement of knowledge, regardless of future applicability” was 3.82. The rating for “health benefit to society (not necessarily in the near future)” was 4.32. The rating for “gain of prestige” was 2.79. The rating for “gain of money” was 2.29. The rating for “satisfaction of their curiosity” was 4.18. The rating for “satisfaction from solving puzzling problems” was 4.16 ( Figure 3B). Thus, these results show that, with the exception of “gain of money”, all other motivations are from “moderately important” to “very important” for more than 50% of the respondents. Moreover, these results show that scientists perceive themselves as more motivated by the pursuit of “health benefit to society (not necessarily in the near future)” and less motivated from the “gain of prestige” and “gain of money” than other scientists.

### Most basic scientists believe it is possible to estimate the potential future health benefits to society from basic biological/biomedical research

**Figure 4.**
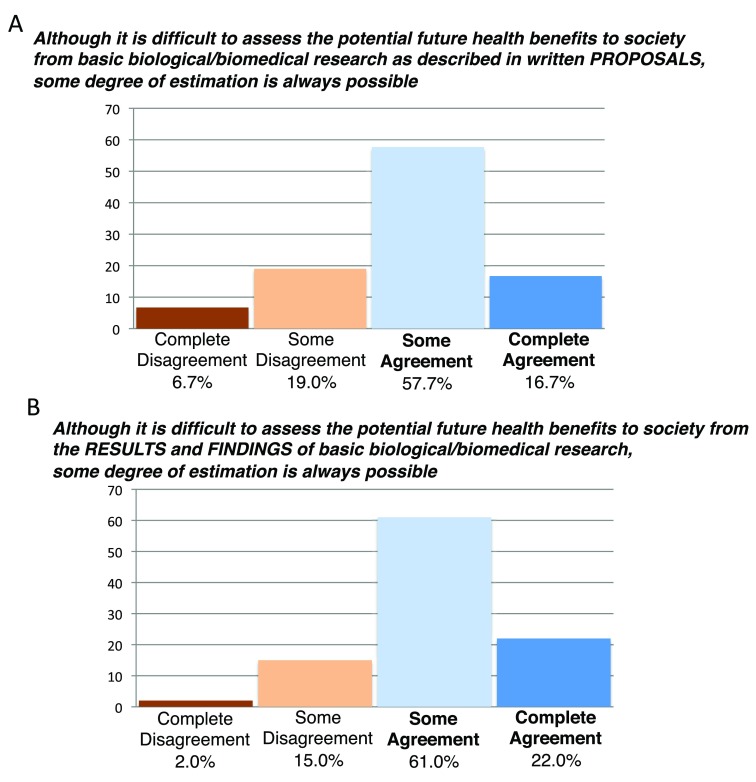
Most scientists think it is possible to estimate future health benefits potential of basic research. ( **A**) Graph shows the levels of agreement on the following statement: “Although it is difficult to assess the potential future health benefits to society from basic biological/biomedical research as described in written PROPOSALS, some degree of estimation is always possible”. 300 scientists answered the question; 4 skipped. ( **B**) Graph shows the levels of agreement on the following statement: “Although it is difficult to assess the potential future health benefits to society from the RESULTS and FINDINGS of basic biological/biomedical research, some degree of estimation is always possible” 300 answered the question; 4 skipped.

To design policies to increase the practical impact of basic biomedical/biological research, it is important to understand whether estimating the health benefit potential of basic research is possible, a topic that has being debated for many years ^11, 27^. We asked respondents to express their level of agreement on scientists’ ability to estimate the potential future health benefits at different stages of the research process. Q11 stated: *“Although it is difficult to assess the potential future health benefits to society from basic biological/biomedical research as described in written PROPOSALS, some degree of estimation is always possible”.* 16.7% of the respondents were in complete agreement with this sentence, 57.7% in some agreement, 19.0% in some disagreement and 6.7% in complete disagreement ( Figure 4A).

Q12 stated: *“Although it is difficult to assess the potential future health benefits to society from the RESULTS and FINDINGS of basic biological/biomedical research, some degree of estimation is always possible”.* 22.0% of the respondents were in complete agreement with this sentence, 61.0% in some agreement, 15.0% in some disagreement and 2.0% in complete disagreement ( Figure 4B). These results therefore show that the majority (83%) of the surveyed scientists believe that estimating the future health benefits to society from the proposals or outcome of basic biological/biomedical projects is somewhat feasible.

### Most basic scientists believe that the discussion of potential medical benefits in basic research proposals is not useful and should be decreased

**Figure 5.**
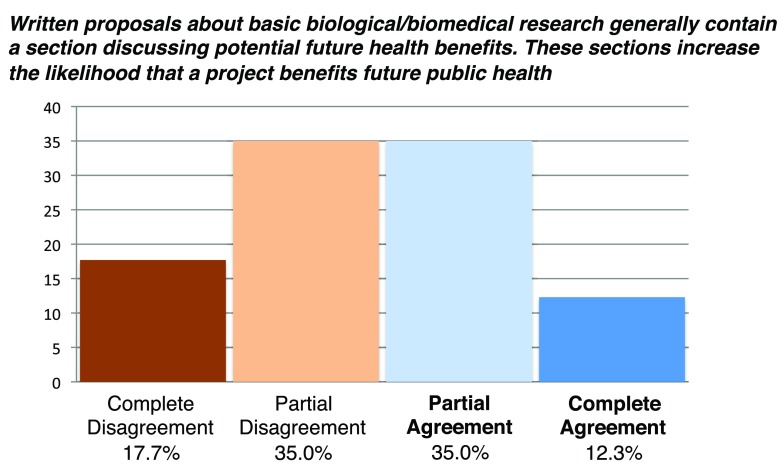
Most scientists think that asking about the health potential (study “significance”) in research proposals is not useful to increase the health potential. Graph shows the levels of agreement on the following statement: “Written proposals about basic biological/biomedical research generally contain a section discussing potential future health benefits. These sections increase the likelihood that a project benefits future public health”. 300 scientists answered the question; 4 skipped.

**Figure 6.**
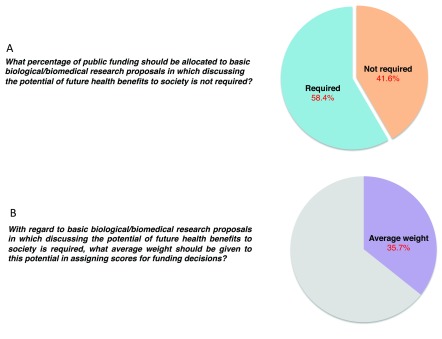
Scientists think more scientific projects should not be asked about their practical outcome potential. ( **A**) Graph shows how surveyed scientists responded to the following question: “What percentage of public funding should be allocated to basic biological/biomedical research proposals in which discussing the potential of future health benefits to society is not required?”. 290 answered the question; 14 skipped. ( **B**) Graph shows how surveyed scientists responded to the following question: “With regard to basic biological/biomedical research proposals in which discussing the potential of future health benefits to society is required, what average weight should be given to this potential in assigning scores for funding decisions?” 285 answered the question; 19 skipped.

**Figure S5.**
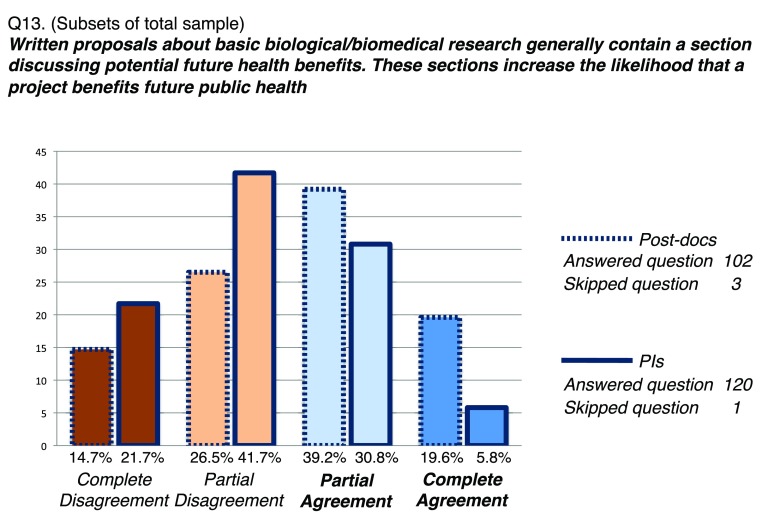
Principal Investigators are sceptical (and more than the post---doctoral researchers) about the effectiveness of discussing potential practical outcome in basic research proposals.

Funding agencies around the world commonly request that the potential health benefits of basic research projects are discussed in the written proposals. To understand what scientists think about this restrictive (i.e. non voluntary) policy, Q13 asked respondents to express their level of agreement with the following statement: *“Written proposals about basic biological/biomedical research generally contain a section discussing potential future health benefits. These sections increase the likelihood that a project benefits future public health”.* 12.3% of the respondents were in complete agreement with this statement, 35.0% were in partial agreement, 35.0% were in partial disagreement and 17.7% were in complete disagreement ( Figure 5). To shed light on how scientists would improve current funding criteria we asked (Q14) the following question: *“What percentage of public funding should be allocated to basic biological/biomedical research proposals in which discussing the potential of future health benefits to society is not required?”* The mean response was that 41.6% of public funding, on average, should be allocated to research in which a discussion of the potential health benefits is not required in written proposals ( Figure 6A) (standard deviation was 25.72; 3.4% of the respondents to this question declared 0%; 6.6% of the respondents declared 100%). Q15 asked: *“With regard to basic biological/biomedical research proposals in which discussing the potential of future health benefits to society is required, what average weight should be given to this potential in assigning scores for funding decisions?”* The average “weight” was 35.7% ( Figure 6B) (standard deviation was 25.87; 6.7% of the respondents to this question declared 0%). Thus, the majority of respondents believe that discussing the potential future health benefits in basic research proposals is not an effective way to increase the likelihood that a project benefits future public health. Principal investigators were substantially more in disagreement than post-docs (63.4% and 41.2%, respectively) with regard to the effectiveness of this policy in increasing societal benefits ( Figure S5). Moreover, these scientists believe that a considerable proportion of public funding (41.6%) should be allocated to research proposals in which discussing the future health benefits to society is not required.

### Most basic scientists are in favor of motivational incentives to increase the likelihood that a research project benefits future public health

**Figure 7.**
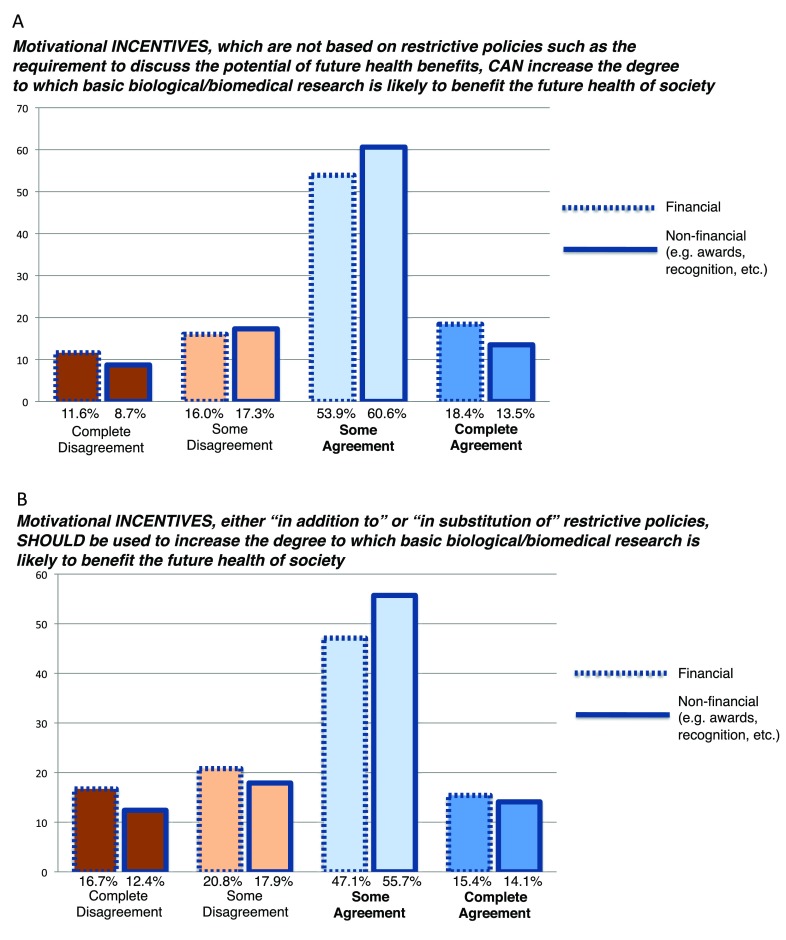
Most scientists are in favor of motivational incentives to increase the health benefit potential of their investigations. ( **A**) Graph shows the levels of agreement on the following statement: “Motivational INCENTIVES, which are not based on restrictive policies such as the requirement to discuss the potential of future health benefits, CAN increase the degree to which basic biological/biomedical research is likely to benefit the future health of society”. 293 answered the question; 11 skipped. The incentives were proposed either as financial or as non-financial. ( **B**) Graph shows the levels of agreement on the following statement: “Motivational INCENTIVES, either “in addition to” or “in substitution of” restrictive policies, SHOULD be used to increase the degree to which basic biological/biomedical research is likely to benefit the future health of society”. 294 answered the question; 10 skipped. The incentives were proposed either as financial or as non-financial.

In order to understand if scientists believe that motivational incentives could be more effective than stricter policies (such as the mandatory discussion of the potential medical benefits in research proposals), we asked (Q16) scientists to express the level of agreement with the following statement: *“Motivational INCENTIVES, which are not based on restrictive policies such as the requirement to discuss the potential of future health benefits, CAN increase the degree to which basic biological/biomedical research is likely to benefit the future health of society”.* With regard to financial incentives, 18.4% of the respondents were in complete agreement with this statement, 53.9% in some agreement, 16.0% in some disagreement and 11.6% in complete disagreement. With regard to non-financial incentives (e.g. awards, recognition), 13.5% of the respondents were in complete agreement with the statement, 60.6% in some agreement, 17.3% in some disagreement and 8.7% in complete disagreement ( Figure 7A).

To understand if motivational incentives should be implemented and used, we asked respondents (Q17) to express the level of agreement on the following slightly different statement: *“Motivational INCENTIVES, either “in addition to” or “in substitution of” restrictive policies, SHOULD be used to increase the degree to which basic biological/biomedical research is likely to benefit the future health of society”.* With regard to financial incentives, 15.4% of the respondents were in compete agreement with this statement, 47.1% in some agreement, 20.8% in some disagreement and 16.7% in complete disagreement. With regard to non-financial incentives, 14.1% of the respondents were in complete agreement with the statement, 55.7% in some agreement, 17.9% in some disagreement and 12.4% in complete disagreement ( Figure 7B).

These results suggest that most responding scientists are in favor of motivational incentives (either financial or non-financial) to be used either “in addition to” or “in substitution of” more restrictive policies to increase the public health potential of basic biological/biomedical research.

### Summary of results

The majority of the scientists who participated in the survey indicated that the most important goal of publicly funded basic “biomedical” research is the production of health benefits to society (86%) ( Figure 2B) and that the desire to effectively benefit society is an important or very important motivation for most of them (87%) ( Figure 3B). While the benefits to society may be not realized in the near future, a substantial majority of respondents (74%) ( Figure 4A) agreed/partially agreed on the idea that some degree of estimation of the potential contribution to human health is possible for every basic research proposal. Further, they indicated that, ideally, more than half of public funding should be allocated to proposals in which a discussion of the potential future health benefits to society is required ( Figure 6A). Moreover, with regard to the definition of basic research, nearly all respondents (94%) ( Figure 1B) indicated that thinking about the future practical benefits of their research is compatible with the status of “basic” researchers, thus implying that basic research should not be conceptualized as (necessarily or solely) driven by curiosity.

Our survey also sheds a light on scientists’ other motivations, besides contributing to the health of society. Such information is useful for the design of incentive-based policies. Our survey confirmed that the so-called “puzzle-motivation”—the satisfaction from solving puzzling problems—was an important motivator ^40^ for almost all basic scientists (among our respondents 93% said that “satisfaction from solving puzzling problems” and 95% that “satisfaction of curiosity” were from “moderately” to “very important” motivations) ( Figure 3B). The so-called “ribbon-motivation”—the gain of prestige and recognition—was substantially more important than the gain of personal money (among our respondents 60% said that the “gain of prestige” was a “moderately” to “very important” motivation for them compared to 41% who said the same for the “gain of money”) ( Figure 3B). Moreover, the majority of respondents of the survey were in favor of using financial incentives (62%) and non-financial incentives (70%) to increase the degree to which basic biological/biomedical research is likely to benefit the future health of society ( Figure 7B).

### Small differences between principal investigators and post-docs responses

To determine if career stage impacted on the responses to the survey, we compared the answers of principal investigators with those of post-docs (see the two new files added to the Data Set) the two most represented groups in our sample. We identified a few differences that are worth mentioning.

Although the percentage of principal investigators and post-docs in agreement with the Q6 statement ( *“Basic scientists can ponder about the future indirect practical benefits of their research without losing their “basic status””*) were similar (96.7% and 94.3%, respectively), the percentage of principal investigators in “complete agreement” was substantially higher than post-docs (80.7% and 64.8%, respectively).

For responses to (Q8) *“YOUR personal motivations as a scientist are from”*, on average principal investigators rated “gain of money (for personal purposes)” as less important than for post-docs (2.00 and 2.58, respectively), indicating that gain of money is a stronger motivator for researchers in the earlier stages of their career than for more senior researchers. Similarly (Q8), the rating average of “gain of prestige” for principal investigators was slightly lower than for post-docs (2.77 and 2.94, respectively), indicating that for younger researchers gain of prestige is a stronger motivator than for older researchers.

When asked (Q9) *“What should the most important goal of publicly funded basic BIOLOGICAL (not biomedical) research be?”* the percent of principal investigators that indicated “health benefit to society (not necessarily in the near future)” was appreciably lower than the share of post-docs (18.1% and 25.0%, respectively). Similarly, when asked (Q10) *“What should the most important goal of publicly funded basic BIOMEDICAL research be?”* the percent of principal investigators that indicated “health benefit to society (not necessarily in the near future)” was lower than the percent of post-docs (81.0% and 86.3%, respectively).

The percent of principal investigators in agreement with the following statement (Q13) *“Written proposals about basic biological/biomedical research generally contain a section discussing potential future health benefits. These sections increase the likelihood that a project benefits public health”* was much lower than the share of post-docs (36.6% and 58.8%, respectively) ( Figure S5).

When asked (Q14) *“What percentage of public funding should be allocated to basic biological/biomedical research proposals in which discussing the potential of future health benefits to society is not required?”* principal investigators and post-docs indicated 44.4% and 40.4%, respectively. Consistent with this, when asked (Q15) *“With regard to basic biological/biomedical research proposals in which discussing the potential of future health benefits to society is required, what average weight should be given to this potential in assigning scores for funding decisions?”* principal investigators and post-docs indicated 31.1% and 39.3%, respectively. Therefore the principal investigators are even move in favor than the post-docs in reducing the extent and the weight of discussions of the future health benefits in research proposals.

Finally, the percent of post-docs in agreement with the use of motivational incentives (Q17) was higher than the percent of principal investigators, both for financial incentives (75.2% and 51.7%, respectively) and for non-financial incentives (71.0% and 67.8%, respectively).

Responses of Harvard Medical School (and affiliate) scientists to the online survey csv file: Responses were collected using Surverymonkey. Responses to question 18 are not included.
pdf file: Full list of questions used in the survey
Principal Investigators: Summaries of responses of principal investigators (new file added to original data set: http://dx.doi.org/10.6084/m9.figshare.902837)
Post docs: Summaries of responses of post-docs (new file added to original data set: http://dx.doi.org/10.6084/m9.figshare.902837)

## Discussion

**Figure 8.**
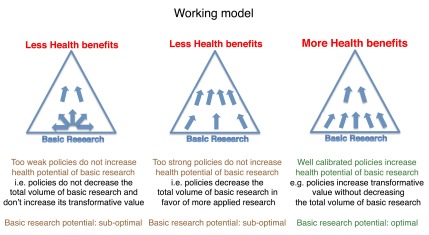
Schematic representation of the principles to increase the health benefit potential of basic biological and biomedical research. In order to increase the health benefits to society from basic “bio” research, policies should produce a good integration between “pure” basic, “use-inspired” basic, translational and applied research. The transformative value of basic science could be increased in several ways (see main text and Box 1 for more details). The directions of the arrows are intended only to express the capacity of transformative value of research (arrows pointing towards the top vertex of the triangle have maximum transformative value) and are not intended to reflect neither the quality of research nor the status of “basicness”.

The results of this survey provide valuable information to help create effective policies to increase the health benefit potential of basic biological and biomedical research and the work satisfaction of scientists without altering the nature and volume of scientific investigations (schematized in Figure 8). Building on these results, we conclude that nonfinancial soft incentives (nudges), in particular, could be valuable tools to maximize the transformative value of basic research. Creative nudges should entail little additional work for scientists and can be implemented without significantly increasing public spending and bureaucratic burden. We also believe that soft incentives would be a valuable departure from some current policies which seem ineffective. For example, while 92% of respondents indicated that they are in favor of an increase in public funding for basic biological/biomedical research ( Figure S4), a substantial majority of the principal investigators (63%) ( Figure S5) declared that the sections in written proposals aimed at discussing the potential future health benefits do not really increase the likelihood that a project will benefit future public health. Our respondents also claimed that more public funding (on average the 42% of the total public funding committed to basic biological/biomedical research) should be devoted to basic biological/biomedical research proposals in which discussing the potential of future health benefits is not required ( Figure 6A).

Based on these findings, we propose a few examples of policies based on soft (sometimes subconscious) incentives that could gently direct some scientists towards undertaking basic research inquiries with higher transformative value ( Box 1). Policies that could be particularly effective are the ones that exploit the scientist’s drive to both achieve a good reputation and to benefit society ^14, 40^. One example would be placing research laboratories inside or in close proximity to hospitals to expose basic scientists to the view of patients and practicing physicians; in this regard it would be useful to know if scientists already working in laboratories inside hospitals have different views, motivations and values than those working in comparable laboratories located far away from hospitals.

Box 1. **Example of nudges (“soft” motivational incentives) potentially useful for increasing the transformative value of basic research without altering its fundamental nature or volume.**

- Locating basic research laboratories inside or in close proximity of hospitals
- Organizing educational and discussion meetings between scientists and the general public or patient associations. Acknowledging the participating scientists. Considering their participations during grant assignments, promotion, hiring etc.
- Organizing more seminars (inside or outside the research institutes) about the purpose of scientific research and scientists in the society. Acknowledging the participating scientists. Considering their participations during grant assignments, promotion, hiring etc.
- Promoting more scholar studies and discussion about the concept and definition of basic research
- Having ethics consultation services inside research institutes. Good information about these services and ease of access and consultation.
- Providing recognition to the basic scientists who have contributed to producing tangible health benefits e.g. requiring a list of seminal basic research articles for each new drug, medical device or other biological applications (see text for more details)
- Promoting a different conceptualization of the notion of basic research (see text for more details)

Another example would be organizing more educational meetings in which scientists explain their work to the general public or to associations of patients. It would probably be wise to keep these meetings non-mandatory and conceive of ways to recognize the participating scientists (e.g. with certificates). This incentive would fit with the increasing interest in blurring the boundaries between the scientific community and the general public discussed above ^21– 23^. A similar nudge would be organizing periodical seminars inside research institutions during which scientists could discuss the role of scientific research and scientists in society. Although non-mandatory, attendance could result in some form of credit or recognition for participation. Another soft incentive could be provided by the presence of ethics consultation offices inside research institutes; a previous study found that most scientists view such a proposal favourably ^37^.

A model of an incentive that exploits the scientist’s aspiration to achieve a good reputation and a role in benefiting public health would be to formally recognize the basic scientists when new drugs or medical devices are approved, as we recently proposed ^41^. This type of incentive would make use of the “ribbon-motivation” but without undermining the “puzzle-motivation” or research freedom generally ^14, 40^. This system could work by implementing a “bibliography of basic papers” for each newly approved drug. To apply this idea, a peer review group would identify the basic papers that have been influential for the development in the drug (or other biological applications) or, alternatively, review a list proposed by the drug owner ^41^. A list of fifty to one hundred basic research papers would be selected and appear in the public databases (like the Orange Book of FDA) and in the drug package. Scientists would receive satisfaction from seeing their work in the list of seminal papers crucial for the design of a new drug, by perceiving their active role in public health improvement and by posting the achievement on their *curriculum vitae* or personal website. This system would be a “weak attractor” because it would not distract scientists from basic research but it would represent a small, mostly unconscious, incentive to pursue research lines that can more easily lead to future drugs and should not create imbalances in the system by pushing basic scientists towards non-basic investigations. Therefore this system would not dramatically affect the whole "ecosystem" of the scientific research that indeed needs to be made of a balanced mix of the different types of research, from the "purely" basic to the "purely" applied ( Figure 8). This method would also present the advantage of increasing public awareness of the role of basic science, which we think is often underestimated by lay people as well as politicians.

Moreover, we believe that a different conceptualization of the notion of basic research would help in increasing the transformative values of fundamental investigations. A portion of basic research should (continue to) be devoted to purely curiosity-driven purposes as knowledge *per se* has a value and increases the quality of life of people through fascination and ‘soul nourishment’. However, basic research should not be conceptualized as solely driven by curiosity. Indeed, in our survey, nearly all respondents (94%) ( Figure 1B) indicated that thinking about the future practical benefits of their research is compatible with the status of “basic” researchers. Therefore, similar to the Organisation for Economic Co-operation and Development (OECD)’s division of the continuum of basic research into pure basic research and oriented basic research ( http://stats.oecd.org/glossary/detail.asp?ID=192), we believe basic research can usefully be divided into two broad categories: solely curiosity-driven research and research driven by a vision of how the knowledge generated might be useful for future applications. In this context, the term “blue skies research”, sometimes used to define the entire field of basic research ^42^ might be used for those studies that are solely (or largely) curiosity-driven. This division of basic research would be similar to the previously proposed division between “pure basic research” (also represented as the “Bohr’s quadrant” as exemplified by the work of the atomic physicist Niels Bohr) and “use-inspired basic research” (also represented as the “Pasteur’s quadrant” as exemplified by the work of the biologist Louis Pasteur) ^11, 43^. Even if curiosity does remain one of the main motivators for conducting and studying science, we believe that basic research should be conceptualized as research that focuses on basic mechanisms of natural phenomena rather than research that is intended to satisfy scientists curiosity (as it is frequently presented in the mass media). Such a mindset would hopefully result in more basic scientists (especially younger ones) pondering the purpose of their research and being inspired by basic research avenues that are “use-inspired”.

We also need to revisit the claim that because the future benefits of basic research cannot be accurately predicted, all basic research is equally valuable, i.e. every imaginable basic investigation would have the same exact potential of practical outcome. In fact, the great majority of scientists who took part in our survey pointed out that, despite the fact that it is usually necessary to undertake a very long pathway (the “countless sources” mentioned by Abraham Flexner ^4^) before being able to funnel basic knowledge toward more applicative studies, some degree of assessment of the transformative value of basic investigations is always possible. It follows that since the potential benefits for society are somewhat predictable, basic research can be evaluated prospectively; this does not lessen the “basic status” neither of the research nor of the scientist. Such a revised mindset could “nudge” more basic scientists (and grant funders) to wonder about the future impact of their investigations. Of course, it will also be important to estimate in the best possible way and, case by case, the degree (i.e. possibility) of assessment of the transformative value, as an overestimation of our ability to assess the transformative value of research projects could have negative effects on both innovation potential and scientist satisfaction. Plausibly, future study and discussion will shed more light on these concepts and increase our ability to assess the social potential of fundamental investigations.

Our survey results show that basic scientists think that the major goal of biomedical research (and one of their highest motivations) is providing health benefits to society (even if not necessarily in the near future). The large majority of respondents were in favor of using soft incentives to increase the health benefit potential of basic biological/biomedical research. The use of “nudges” seems to be particularly promising with the basic scientists at the earlier stages of their career; compared to principal investigators post-docs are more likely to think that the major goal of basic biological and biomedical research is to provide health benefits to society, are even more driven by prestige and financial motivations and are even more in favor of the use of soft incentives. This suggests that in the near future even more scientists will be suitable for soft motivational incentives, even if we cannot exclude that current younger scientists will slightly change their perspectives and values when they reach more advanced stages in their career. Even so, most principal investigators are also in favor of using soft incentives (especially non-financial ones) and are dissatisfied (substantially more so than the post-docs ( Figure S5)) with the current policies requiring the assessment of potential health benefit.

Our study has various limitations. For example, it may not be generalizable, since it involves only scientists working at Harvard Medical School and affiliated Boston-area institutions. For this reason, it would be useful to sample other scientific communities (i.e. those operating in different geographical and cultural contexts). Moreover, in order to implement effective incentives, it would be important to analyze in depth the scientist’s perception about the specific policies ( Box 1). Furthermore, it would be strategic to collect more data to coordinate the implementation of these incentives with the improvement of current policies regulating research evaluation and funding assignments.

Basic research advances knowledge that, regardless of its possible practical outcome, has a value *per se*. In addition, basic research has also the potential to produce more practical benefits to humanity, such as the prevention and treatment of diseases. As a society, we have the moral obligation to try to maximize this potential. We believe, and the data presented in the paper support, that soft incentives can be valuable tools for increasing this potential without corrupting the spirit of fundamental investigations, thus further aligning the goals of cell and molecular biologists with those of the broader public health community.

## Methods

Ethics statement: On April 2, 2012, the Institutional Review Board (IRB) of Harvard School of Public Health determined that the proposed study meets the criteria for exemption per the regulations found at 45 CFR 46.101(b) ^2^. The IRB made the following determinations: Research Information Security Level; the research is classified, using Harvard’s Data Security Policy, as Level 1 data. The notification was signed by QA/QI specialist.

The survey was designed as an online questionnaire (powered by SurveyMonkey, www.surveymonkey.com) made of 17 questions (Q1–Q17) plus one additional field for free comments (separate manuscript, submitted and under revision). Answers could be provided through multiple choices or, alternatively, textboxes for alphanumerical entries. Each question had the option to be skipped. The survey was sent to a sample of scientists involved in basic biological/biomedical studies (for the most part, cell and molecular biology studies). The scientists were also asked to confirm their level of involvement in basic fundamental research (see results section). The responses were collected during 9 consecutive weeks during 2012. Principal investigator (PI) scientists were contacted by email after consulting the websites of Harvard University and some affiliated institutes (Brigham and Women’s Hospital, Beth Israel Deaconess Medical Center, Dana-Farber Cancer Institute, Joslin Diabetes Center and Children’s Hospital); the majority of principal investigators were asked to forward the survey to members of their own groups. Post-docs were contacted either by their PIs or by using university-associated mailing lists and networking. Also a few scientists with other types of position (e.g. PhD students, instructors, research assistants) took part in the survey, generally contacted by their PIs. In addition to the specific request to forward the survey to their own groups or to close intra-institutional colleagues, the contacted scientists were specifically asked not to forward the survey to the outside community. The survey was completely voluntary. The response rate is not known because we do not know how many scientists actually read the invitation email and how many principal investigators forwarded the invitation to their lab members. We used this approach because we wanted to maximize the sample size. By taking in consideration only the scientists that took part to the survey, the response rate was very high for all questions (on average, fewer than 2% of the respondents, with a range from 0% (Q2 and Q3) to 6.3% (Q15) skipped any of the 17 questions); this suggests that the survey did not contain difficult-to-understand or difficult-to-answer questions. Therefore the decision of participating (or not) in the survey was probably not based on the nature of the questions but rather on other factors (e.g. lack of time) that, conceivably, have only a marginal effect on the representativity of the sample. Moreover, the survey was completely anonymous. For these reasons, we believe that the sample of scientists that took part to this survey is fairly representative of the entire population of scientists working in the same setting (Harvard Medical School and affiliated institutes).

## Data availability

figshare: Responses of Harvard Medical School (and affiliate) scientists to the online survey, doi: 10.6084/m9.figshare.1036485 ^44^
