# Supplementary material for: Policies to increase the social value of science and the scientist satisfaction. An exploratory survey among Harvard bioscientists
Source: F1000Res. 2014 Jun 6;3:20. Originally published 2014 Jan 21. [Version 2] doi: 10.12688/f1000research.3-20.v2 (PMC3999931; doi:10.12688/f1000research.3-20.v2)
Supplement: Responses of Harvard Medical School (and affiliate) scientists to the online survey — csv file: Responses were collected using Surverymonkey. Responses to question 18 are not included. pdf file: Full list of questions used in the survey Principal Investigators: Summaries of responses of principal investigators (new file added to original data set: http://dx.doi.org/10.6084/m9.figshare.902837) Post docs: Summaries of responses of post-docs (new file added to original data set: http://dx.doi.org/10.6084/m9.figshare.902837) [file f1000research-3-4604-s0000.tgz › full_questionnaire.pdf]

Basic Research Goals

Exit this survey

This short questionnaire is for a research study conducted at the Department of Health Policy and Management of the Harvard School of Public Health.

It is being sent to a selected sample of scientists working in biological/biomedical research. It is anonymous and contains 18 questions. Participation is completely voluntary. You can skip questions that make you feel uncomfortable. Please do not share the survey web link.

If you have any questions about this survey you can contact Dr. Andrea Ballabeni at aballab@hsph.harvard.edu or Dr. David Hemenway at hemenway@hsph.harvard.edu

Your input is very important to us.
Thank you very much for your time.

1. Please indicate which of the following best describes you:

- ○ Principal investigator
- ○ Post-doc
- ○ PhD student
- ○ Other (please specify) __________

2. Are you?

- ○ Female
- ○ Male

3. Approximately, what percentage of your research do you consider to be basic?

Percentage [0-100] _____

4. Despite the current economic situation, public funding for basic biological/biomedical research should be increased.

- ○ I Agree
- ○ I Disagree

5. Basic research can be defined as the research that is not intended to yield immediate

practical benefits except for advancement of knowledge.

- ◯ Complete disagreement
- ◯ Some disagreement
- ◯ Some agreement
- ◯ Complete agreement

6. Basic scientists can ponder about the future indirect practical benefits of their research without losing their "basic status".

- ◯ Complete disagreement
- ◯ Some disagreement
- ◯ Some agreement
- ◯ Complete agreement

7. The motivations of MOST basic biological/biomedical scientists are from:

	Not a motivation	Minimally important	Moderately important	Important	Very important
Pure advancement of knowledge, regardless of future applicability	◯	◯	◯	◯	◯
Health benefit to society (not necessarily in the near future)	◯	◯	◯	◯	◯
Gain of prestige	◯	◯	◯	◯	◯
Gain of money (for personal purposes)	◯	◯	◯	◯	◯
Satisfaction of their curiosity	◯	◯	◯	◯	◯
Satisfaction from solving puzzling problems	◯	◯	◯	◯	◯

8. YOUR personal motivations as a scientist are from:

	Not a motivation	Minimally important	Moderately important	Important	Very important
Pure advancement of knowledge, regardless of future applicability	◯	◯	◯	◯	◯
Health benefit to society (not necessarily in the near future)	◯	◯	◯	◯	◯

Gain of prestige	◯	◯	◯	◯	◯
Gain of money (for personal purposes)	◯	◯	◯	◯	◯
Satisfaction of your curiosity	◯	◯	◯	◯	◯
Satisfaction from solving puzzling problems	◯	◯	◯	◯	◯

9. What should the most important goal of publicly funded basic BIOLOGICAL (not biomedical) research be?

- ◯ Health benefit to society (not necessarily in the near future)
- ◯ Pure advancement of knowledge, regardless of future applicability
- ◯ Other (please specify)

10. What should the most important goal of publicly funded basic BIOMEDICAL research be?

- ◯ Health benefit to society (not necessarily in the near future)
- ◯ Pure advancement of knowledge, regardless of future applicability
- ◯ Other (please specify)

11. Although it is difficult to assess the potential future health benefits to society from basic biological/biomedical research as described in written PROPOSALS, some degree of estimation is always possible.

◯ Complete disagreement ◯ Some disagreement ◯ Some agreement ◯ Complete agreement

12. Although it is difficult to assess the potential future health benefits to society from the RESULTS and FINDINGS of basic biological/biomedical research, some degree of estimation is always possible.

◯ Complete disagreement ◯ Some disagreement ◯ Some agreement ◯ Complete agreement

13. Written proposals about basic biological/biomedical research generally contain a

section discussing potential future health benefits. These sections increase the likelihood that a project benefits future public health.

○ Complete disagreement ○ Partial disagreement ○ Partial agreement ○ Complete agreement

14. What percentage of public funding should be allocated to basic biological/biomedical research proposals in which discussing the potential of future health benefits to society is not required?

Percentage [0-100] [ ]

15. With regard to basic biological/biomedical research proposals in which discussing the potential of future health benefits to society is required, what average weight should be given to this potential in assigning scores for funding decisions?
[You can enter 0 only if your answer to previous question is 100]

Percentage [0-100] [ ]

16. Motivational INCENTIVES, which are not based on restrictive policies such as the requirement to discuss the potential of future health benefits, CAN increase the degree to which basic biological/biomedical research is likely to benefit the future health of society.

	Complete disagreement	Some disagreement	Some agreement	Complete agreement
Financial incentives	○	○	○	○
Non-financial incentives (e.g. awards, recognition, etc.)	○	○	○	○

17. Motivational INCENTIVES, either "in addition to" or "in substitution of" restrictive policies, SHOULD be used to increase the degree to which basic biological/biomedical research is likely to benefit the future health of society.

	Complete disagreement	Some disagreement	Some agreement	Complete agreement
Financial incentives	○	○	○	○
Non-financial incentives (e.g. awards, recognition, etc.)	○	○	○	○

18. Do you have any ideas about possible INCENTIVES to motivate basic biological/biomedical scientists to do more of the basic research that is more likely to benefit public health in the future?
You can use this space to tell us about them or for any additional considerations related to this survey.

Thank you for completing this survey and helping our research!

Done

Powered by **SurveyMonkey**
Check out our sample surveys and create your own now!
