# Supplementary material for: Policies to increase the social value of science and the scientist satisfaction. An exploratory survey among Harvard bioscientists
Source: F1000Res. 2014 Jun 6;3:20. Originally published 2014 Jan 21. [Version 2] doi: 10.12688/f1000research.3-20.v2 (PMC3999931; doi:10.12688/f1000research.3-20.v2)
Supplement: Peer review history

# Peer review: Referee response for version 2

**DOI:** 10.5256/f1000research.4604.r5040

**Published:** 2014-06-17

**Authors:** Daniel Strech

**Affiliations:** 1 CELLS - Centre for Ethics and Law in the Life Science Institute for History, Ethics and Philosophy of Medicine, Hannover Medical School, Hannover, Germany

The revisions improved the paper. I have no further comments.

I have read this submission. I believe that I have an appropriate level of expertise to confirm that it is of an acceptable scientific standard.

# Peer review: Referee response for version 1

**DOI:** 10.5256/f1000research.3266.r4134

**Published:** 2014-03-31

**Authors:** Daniel Strech

**Affiliations:** 1 CELLS - Centre for Ethics and Law in the Life Science Institute for History, Ethics and Philosophy of Medicine, Hannover Medical School, Hannover, Germany

This study addresses an interesting and relevant topic. Box 1 in particular is innovative and makes a good link to the findings.

I have the following comments:

1. More remarks are needed about the relevant body of evidence. Are there other studies that investigated the self-understanding of basic scientists in biomedicine? The Background mentions none and the Discussion only one paper.
2. The main text should make clear that the response rate and reasons for non-response are not known. A brief explanation together with a justification on why this approach was selected should be given.
3. Figure 6 is somewhat simplistic and packed with several unjustified assumptions on what the (true) positive and negative effects of specific pattern of resource allocation to basic or translational research are. This should be deleted. The legend to figure 6 is very general, for example the recommendation saying that one should “ *produce a good integration between basic and applied investigations …*”.

I have read this submission. I believe that I have an appropriate level of expertise to confirm that it is of an acceptable scientific standard.

# Response

**Published:** 2014-06-03

**Authors:** Andrea Ballabeni

**Affiliations:** Harvard School of Public Health, USA

We would like to thank Dr Daniel Strech for his helpful feedback and for giving us the possibility to improve our manuscript. We have tried to address each of his comments. We provide below our answers point-by-point.

*- More remarks are needed about the relevant body of evidence. Are there other studies that investigated the self-understanding of basic scientists in biomedicine?*

**We are really grateful for the excellent advice of providing more references (and connected remarks) about the relevant body of knowledge. We have therefore added 28 new references and related new remarks in the Introduction and in the Discussion sections.**

*- The main text should make clear that the response rate and reasons for non-response are not known. A brief explanation together with a justification on why this approach was selected should be given.*

**We agree with the referee that this is an important thing to discuss in the manuscript. Therefore we have added a discussion in the Methods section:**

**“ *The response rate is not known because we do not know how many scientists actually read the invitation email and how many principal investigators forwarded the invitation to their lab members. We used this approach because we wanted to maximize the sample size. By taking in consideration only the scientists that took part to the survey, the response rate was very high for all the questions (on average, fewer than 2% of the respondents, with a range from 0% (Q2 and Q3) to 6.3% (Q15) skipped any of the 17 questions); this suggests that the survey did not contain difficult-to-understand or difficult-to-answer questions. Therefore the decision of participating (or not) to the survey was probably not based on the nature of the questions but rather on other factors (e.g. lack of time) that, conceivably, have only a marginal effect on the representativity of the sample. Moreover, the survey was completely anonymous. For these reasons, we believe that the sample of scientists that took part to this survey is fairly representative of the entire population of scientists working in the same setting (Harvard Medical School and affiliated institutes).”***

*- Figure 6 is somewhat simplistic and packed with several unjustified assumptions on what the (true) positive and negative effects of specific pattern of resource allocation to basic or translational research are. This should be deleted. The legend to figure 6 is very general, for example the recommendation saying that one should “produce a good integration between basic and applied investigations …”.*

**We thank the referee for this comment. While figure 6 (now figure 8) may look simplistic and general, this design is deliberate because we think that the simple schematizations contained in it help the readers visualize and fully grasp some of the concepts discussed in the manuscript. For these reasons, we would like to keep this figure. However, we have slightly edited the figure legend and the figure itself in order to further emphasize the concept that we are just showing a schematic ‘working model’ (and like all working models, it may contain assumptions and generalizations).**

# Peer review: Referee response for version 1

**DOI:** 10.5256/f1000research.3266.r3242

**Published:** 2014-01-30

**Authors:** Giorgio Scita

**Affiliations:** 1 IFOM, FIRC Institute of Molecular Oncology Foundation at IFOM-IEO Campus, Milan, Italy

This is an interesting well-conducted study that clarifies the way basic science is perceived by basic scientists.

It would have been interesting to assess whether the career stage of the respondents has any impact on their answers to the survey.

In the discussion a point is raised as to whether there is the need to revisit “ *the idea that since the future benefits of basic research cannot be accurately predicted, all basic research is equally valuable*” based on the finding that the majority of scientists believe that “ *some degree of assessment of the transformative value of basic investigations is always possible*”. However in the absence of a clear definition of what the degree of assessment is, it remains difficult to fully support such a perception. Thus caution should be exerted in drawing a conclusion as to whether this mindset could “nudge” more basic scientists (and grant funders) to wonder about the future impact of their investigations.

The list of soft motivational incentives in BOX1 is thought provoking and stimulating.

I have read this submission. I believe that I have an appropriate level of expertise to confirm that it is of an acceptable scientific standard.

# Response

**Published:** 2014-06-03

**Authors:** Andrea Ballabeni

**Affiliations:** Harvard School of Public Health, USA

We would like to thank Dr Giorgio Scita for his helpful feedback and for giving us the possibility to improve our manuscript. We have tried to address each of his comments. We provide below our answers point-by-point.

*-  It would have been interesting to assess whether the career stage of the respondents has any impact on their answers to the survey.*

**To address this comment we have created a new paragraph in the Results section in which we list the most meaningful differences between the answers of the principal investigators and the answers of the post-docs. In general, there were not huge differences. However we believe that a few differences are noteworthy.**

**We have also added a few sentences in the Discussion section in order to comment about these differences e.g. “ *… The use of “nudges” seems to be particularly promising with the basic scientists at the earlier stages of their career; compared to principal investigators, post-docs are more likely to think that the major goal of basic biological and biomedical research is to provide health benefits to society, are even more driven by prestige and financial motivations and are even more in favor of the use of soft incentives…*”.**

**We have also submitted the summaries of all the answers of the principal investigators and all the answers of the post-docs; we propose to insert two links into the main text to enable access to these two summaries.**

*- In the discussion a point is raised as to whether there is the need to revisit “the idea that since the future benefits of basic research cannot be accurately predicted, all basic research is equally valuable” based on the finding that the majority of scientists believe that “some degree of assessment of the transformative value of basic investigations is always possible”. However in the absence of a clear definition of what the degree of assessment is, it remains difficult to fully support such a perception. Thus caution should be exerted in drawing a conclusion as to whether this mindset could “nudge” more basic scientists (and grant funders) to wonder about the future impact of their investigations.*

**The referee is right about the absence of a clear definition of what a “degree of assessment” is. For this reason we have added some thoughts in the Discussion section: “ *Of course, it will also be important to estimate in the best possible way and case by case, the degree (i.e. possibility) of assessment of the transformative value as an overestimation of our ability to assess the transformative value of research projects could have negative effects on both the innovation potential and the scientist satisfaction. Plausibly, future study and discussion will shed more light on these concepts and increase our ability to assess the social potential of fundamental investigations*.”**

**We hope that this addition will fully address the referee comment.**
